# Mongolia Red Cross Society, influenza preparedness planning and the response to COVID-19: the case for investing in epidemic preparedness

**DOI:** 10.5365/wpsar.2020.11.2.013

**Published:** 2020-11-13

**Authors:** Lisa Natoli, Narangoo Gaysuren, Densmaa Odkhuu, Veronica Bell

**Affiliations:** a Australian Red Cross, Melbourne, Australia.; b Mongolian Red Cross Society, Ulaanbaatar, Mongolia.

Globally, seasonal influenza contributes to approximately 291 000 to 645 000 deaths each year. (*1*) The burden of annual influenza epidemics can be particularly high in low- and middle-income countries (*1*) such as Mongolia, (*2*) which experienced a nationwide epidemic of influenza A(H1N1) 2009 in the winter of 2018–2019. The national health system was quickly overwhelmed, prompting the State Emergency Commission to involve key partners – including the Mongolia Red Cross Society (MRCS) and its network of more than 6000 volunteers – to augment the Government’s response capacity. This paper describes how the experience of MRCS and subsequent planning for the 2019–2020 influenza season were effectively leveraged during the response to coronavirus disease 2019 (COVID-19).

Over the last decade, MRCS has been engaged by the Government to support the prevention and control of several communicable disease outbreaks; however, MRCS had had little involvement in influenza-related activities since the 2009 H1N1 pandemic. Recognizing the value of preparedness after the winter of 2018–2019, MRCS developed an influenza preparedness plan in advance of the 2019–2020 influenza season. Planning comprised a review of seasonal influenza risk, including risk factors and vulnerability; mapping key stakeholders and relevant policies, plans and capacities; and a literature review to determine the evidence base for community-focused, influenza-related interventions.

Aligned with recommendations of the WHO *Global Influenza Strategy 2019–2030* (*3*) and structured around the “epidemic response cycle” (preparedness, alert, response and evaluation), (*4*) the preparedness plan set out actions for MRCS to contribute to mitigating the threat of seasonal influenza, including annual training of volunteers, pre-positioning of health communication materials and hand sanitizer, and strengthening planning and collaboration with local authorities and stakeholders. Volunteer training and activities focused on non-pharmacological interventions – strategies individuals or communities could adopt when well (to reduce exposure to the virus and avoid infection) or unwell (to avoid spreading the infection to others). (*5*) Prevention messages focused on hand hygiene, cleaning of high-touch surfaces, respiratory etiquette, self-quarantine when feeling unwell and promoting annual influenza vaccination, especially for children aged 2–5 years. Tailored education messages for children were delivered through games, songs and fun activities rather than didactic approaches.

The preparedness plan aimed to strengthen community-centred preparedness for seasonal influenza, as well as to provide the foundation for MRCS to contribute to broader epidemic preparedness. The benefits of this were almost immediate, with MRCS leveraging the plan in the preparedness phase of the national COVID-19 response in January 2020. Many of MRCS’s routine influenza prevention messages similarly apply to COVID-19, and they were quickly rebranded for this purpose and incorporated into volunteer training and public communication materials. Relationships established with national and local authorities to address seasonal influenza were swiftly capitalized upon to enable joint planning and information sharing for COVID-19. And continuous reflection and evaluation processes are providing lessons learnt to inform ongoing COVID-19 interventions, as well as preparedness actions for the 2020–2021 influenza season. For example, a public survey to determine communication preferences highlighted gaps in the MRCS approach, and it has resulted in greater use of television and radio to better reach herder communities.

The COVID-19 pandemic underscores the importance and value of investing in epidemic preparedness planning well in advance of disease outbreaks. Thus far, Mongolia has effectively contained COVID-19 through a proactive and comprehensive public health response that acknowledges and values the role of the community and community-based organizations in health promotion and disease prevention. (*6*) As of 30 August 2020, Mongolia had reported 301 cases of COVID-19 and no community transmission. (*7*) As we have seen with other epidemics, (*8*) community volunteers working to advance health literacy can play a vital role in epidemic disease prevention, detection and response. However, it takes time and resourcing to train and equip them with the necessary skills and communication materials, and for them to gain the trust and respect of their community peers and build necessary credibility to be listened to when outbreaks occur. (*9*) Similarly, strong organizational partnerships do not develop overnight, but once in place they can be effectively leveraged when emergencies occur. The partnership between MRCS and the Ministry of Health established through the influenza experience resulted in greater recognition of the organization’s epidemic preparedness and response “value add” through its vast volunteer network – and ultimately in MRCS being assigned responsibility for community-level health communication and psychosocial support in the COVID-19 response. To date more than 2000 volunteers have shared prevention messages and provided reassurance and support to more than 290 000 people, and assisted more than 7000 repatriated nationals in home quarantine (which occurs for several weeks following centralized quarantine in government facilities); support to those in home quarantine includes two social welfare checks per week providing social connection and reassurance, practical help (for example, grocery shopping), reinforcement of prevention messages and referral to formal mental health services if needed.

The COVID-19 pandemic is a reminder of the need to intensify and sustain the commitment to public health preparedness. (*10*) Well prepared communities, empowered to take action when a threat is detected, are critical to determining if health risks escalate from a local containable outbreak to national and regional threats. The return on investment is incontestable. (*11*)
